# Effectiveness of artificial intelligence chatbots on mental health & well-being in college students: a rapid systematic review

**DOI:** 10.3389/fpsyt.2025.1621768

**Published:** 2025-10-21

**Authors:** Shahzadhi Nyakhar, Hongwu Wang

**Affiliations:** ^1^ Department of Social & Behavioral Sciences, College of Public Health and Health Professions, University of Florida, Gainesville, FL,, United States; ^2^ Department of Occupational Therapy, College of Public Health and Health Professions, University of Florida, Gainesville, FL,, United States

**Keywords:** mental health, artificial intelligence, chatbot, conversational agent, college students

## Abstract

**Background:**

Mental health disorders among college students have surged in recent years, exacerbated by barriers such as stigma, cost associated with treatment, and limited access to mental health providers. Artificial intelligence (AI)-driven chatbots have emerged as scalable, stigma-free tools to deliver evidence-based mental health support, yet their efficacy specifically for college populations remains underexplored.

**Objective:**

This systematic rapid review evaluates the effectiveness of chatbots in improving mental health outcomes (e.g., anxiety, depression) and well-being among college students while identifying key design features and implementation barriers.

**Methods:**

Four databases (PubMed, PsycInfo, Applied Science & Technology Source, ACM Digital Library) were searched for studies published between 2014 and 2024. Two reviewers independently screened articles using predefined PICO criteria, extracted data and assessed quality via the PEDro scale. Included studies focused on chatbot interventions targeting DSM-5-defined mental health conditions or well-being in college students.

**Results:**

Nine studies (n=1,082 participants) were included, with eight reported statistically significant improvements in anxiety (e.g., GAD-7 reductions), depression (e.g., PHQ-9 scores), or well-being. Effective chatbots frequently incorporated cognitive-behavioral therapy (CBT), daily interactions, and cultural personalization (e.g., 22% depression reduction with Woebot; p<0.05). However, heterogeneity in study quality (PEDro scores: 1–7), high attrition rates (up to 61%), and reliance on self-reported outcomes limited generalizability.

**Conclusions:**

Though the use of chatbots for the improvement of mental health and well-being is promising based on the review’s results, future research should prioritize rigorous RCTs, standardized outcome measures (e.g., PHQ-9, GAD-7), and strategies to improve attrition.

## 1 Introduction

The transition to college represents a critical developmental period marked by academic, social, and financial stressors, which can significantly impact mental health. According to the American College Health Association (1), approximately 80% of college students report feeling overwhelmed by their responsibilities, while 75% lack access to adequate mental health services (2). These challenges were exacerbated by the COVID-19 pandemic, which triggered unprecedented disruptions to campus life. A large-scale survey of over 45,000 undergraduate and graduate students revealed that 35% met the criteria for major depressive disorder and 39% for generalized anxiety disorder post-pandemic (3). Contributing factors included social isolation from remote learning, health-related fears, financial instability, and abrupt lifestyle changes (4). Despite this growing need, systemic barriers to traditional mental health care, such as counseling, exist. According to Ebert et al. (5), stigma is a critical barrier among college students accessing mental health care and many would rather seek self-help. Other barriers include high treatment cost, limited availability of providers, and long waiting periods (6, 7). These barriers leave many college students seeking mental health care without timely support.

To address these gaps, scalable and accessible interventions are urgently needed. Artificial intelligence (AI)-driven chatbots have emerged as a promising solution, offering 24/7 availability, anonymity to reduce stigma, and low-cost delivery of evidence-based strategies such as cognitive-behavioral techniques (CBT) and mindfulness (8–10). Preliminary studies suggest chatbots may improve emotional well-being by providing psychoeducation, mood tracking, and coping skill development (2, 11). The use of psychological theories like CBT, which targets maladaptive thought patterns, chatbots provide structured interventions, including mood tracking, psychoeducation, and coping skill development (12). Their conversational nature fosters therapeutic alliance, mimicking aspects of human interaction while remaining scalable (13, 14). Unlike traditional telehealth, chatbots can deliver consistent, tailored support without requiring extensive infrastructure, making them particularly suitable for college settings (15). However, there is a lack of reviews focused solely on college students, and the evidence remains fragmented, with variability in chatbot design (e.g., rule-based vs. AI-driven), target outcomes (e.g., anxiety reduction vs. general well-being), and methodological rigor. Additionally, limited research explores barriers to engagement, such as privacy concerns or user preferences for human interaction (6).

This systematic rapid review aims to synthesize existing evidence on the effectiveness of chatbots in improving mental health and well-being among college students. “Mental health” is operationalized using DSM-5 diagnostic criteria, validated measures of negative affect (e.g., PHQ-9 for depression), and subjective well-being scales. Secondary objectives include identifying (1) barriers to chatbot adoption, (2) design features linked to efficacy, and (3) gaps in current research. The study addresses the question: What evidence exists regarding the effectiveness of chatbots in improving mental health outcomes and well-being in college students, and what factors influence their implementation?

The decision to conduct a rapid review was driven by the need to synthesize emerging evidence on this topic in a timely manner to inform ongoing interventions. Given the evolving nature of the subject and its relevance to current priorities, as well as lack of randomized controlled trials, we aimed to provide a concise, evidence-informed synthesis that could be accessible within a shorter time frame. We would also like to note the small number of included studies reflects the current state of the literature rather than a limitation of our search strategy or review process. These were the only studies that met our pre-defined inclusion criteria based on study quality, relevance, and methodological rigor. A full systematic review would have yielded the same set of studies, but would have required considerably more time and resources without changing the conclusions.

By evaluating chatbots’ potential to bridge mental health care gaps, this review informs universities, developers, and policymakers seeking cost-effective solutions. Findings will highlight best practices for integrating chatbots into campus wellness programs while addressing limitations (e.g., ethical concerns, cultural responsiveness) to ensure equitable access.

## 2 Methods

This systematic rapid review followed established guidelines for accelerated evidence synthesis (16), retaining core systematic review principles (17) to minimize bias while streamlining processes to meet time constraints. Key adaptations included focused search strategies, predefined PICO criteria, and single-reviewer title/abstract screening with dual verification. A health science librarian at the University of Florida collaborated on search term development and database selection to optimize precision and recall.

### 2.1 Study selection

#### 2.1.1 Information sources

Four electronic databases were queried on June 18, 2024: PubMed (biomedical literature), PsycInfo (psychological sciences), Applied Science & Technology Source (technology applications), and ACM Digital Library (computer science). This combination of databases provides a balanced, interdisciplinary approach, covering medical, psychological, technological, and computational perspectives. The search strategy adhered to the Population, Intervention, Comparator, Outcomes (PICO) framework (**Table 1**) and combined controlled vocabulary (e.g., MeSH terms) with free-text keywords. Filters included English-language full-text articles published between 2014–2024 to capture advancements in AI-driven chatbots post-2014. Limiting the review to the last decade ensures relevance, as AI chatbot technology has rapidly evolved during this period. This timeframe captures recent advancements and their applications in mental health, aligning with current technological and academic trends.

**Table 1:** Proposed inclusion and exclusion criteria for screening and selecting studies.

PICO Framework	Inclusion criteria	Exclusion criteria
Population	The population includes all college students, both undergraduate and graduate.	The review will exclude any articles or interventions that look at using chatbots for any individuals outside of the university setting.
Inclusion	The review will include any articles that use chatbots as a form of treatment intervention for mental health disorders and well-being.	The review will exclude any articles where chatbots were not used for treatment purposes (for example- chatbots to diagnose a condition, or to improve clinic flow will be excluded).
Exclusion	Not applicable.	
Outcome	The review will explore the effectiveness chatbots have in treating any mental health disorder as classified in the Diagnostic and Statistical Manual of Mental Disorders (DSM-5-TR) or well-being.	The review will exclude articles that attempt to use chatbots as a treatment for disorders outside of mental health disorders classified in the Diagnostic and Statistical Manual of Mental Disorders (DSM-5-TR) or well-being.

#### 2.1.2 Search strategies

The Boolean syntax integrated three domains:

Mental health:
 (“mental disorder*” OR “mood disorder*” OR depression OR “anxiety disorder*” OR “DSM-5” OR “well-being”).
Intervention:
 (“chatbot*” OR “conversational agent*” OR “virtual assistant*”).
Population:
 (“college student*” OR “university student*” OR “undergraduate*” OR “graduate student*”).

#### 2.1.3 Eligibility criteria

Studies were included if they: Evaluated chatbots as treatment interventions (e.g., CBT delivery, mood tracking) for mental health disorders (DSM-5-defined) or well-being. Focused on college students (undergraduate/graduate). Reported quantitative or qualitative outcomes (e.g., PHQ-9 scores, user satisfaction). Exclusion criteria: Non-treatment applications (e.g., diagnostic tools), non-college populations, non-English texts, and gray literature.

Covidence systematic review software (Veritas Health Innovation, Melbourne, Australia) was used to screen and select literature. The literature screening process consisted of two phases: the title and abstract screening and the full-text screening.

During the title/abstract screening phase, the relevance of each study based on the information in the title and abstract was evaluated by a single first reviewer and one other independent second reviewer. Studies meeting the pre-established inclusion criteria or those requiring further assessment based on the information provided proceeded to the full-text screening phase. In the full-text screening phase, each eligible study underwent a thorough examination to determine its suitability for inclusion in the review. Any discrepancies between the reviewers’ assessments were resolved through consensus discussions. This rigorous screening process ensured the selection of studies that met the established criteria and contributed relevant data to the review.

### 2.2 Data extraction and screening

Data extraction was carried out using a standardized extraction form by two reviewers. Extraction of information included: Article title, publication year, research purpose, research design, setting or data source, participant recruitment, participant eligibility criteria, study participant characteristics, statistical analyses on interested outcomes, and key findings. The extracted findings on changes in mental health or well-being were narratively synthesized to identify the effectiveness of a chatbot on a college student’s mental health/well-being.

#### 2.2.1 Data synthesis and analysis

Extracted data were thematically grouped by Intervention type: Cognitive-behavioral therapy (CBT), mindfulness, and crisis support. Outcomes: Symptom reduction (e.g., depression/anxiety scores), engagement metrics (e.g., usage frequency), and user acceptability. Barriers: Privacy concerns, technical limitations, cultural relevance.

### 2.3 Quality appraisal

Each included study was assessed for its level of evidence using the guidelines from the John’s Hopkins Evidence-Based Practice Model and PEDro scale. John’s Hopkins Evidence-Based Practice Model assigns the study a level of evidence based on its design. Randomized controlled trials are Level 1, quasi-experimental studies are Level 2, experimental studies or systematic reviews are Level 3, and opinion-based studies are classified as Level 4 (18). The PEDro scale measures the validity of randomized and clinical trials (19). There is a set of 11 different criteria and the criteria are “scored” at the end. A score of <4 indicates poor overall quality of judgment, 4–5 indicates fair overall quality of judgment, 6–8 indicates good overall quality of judgment, and 9–10 indicates good excellent quality of judgment. Two reviewers independently appraised studies; discrepancies were resolved through discussion.

## 3 Results

**Figure 1:**
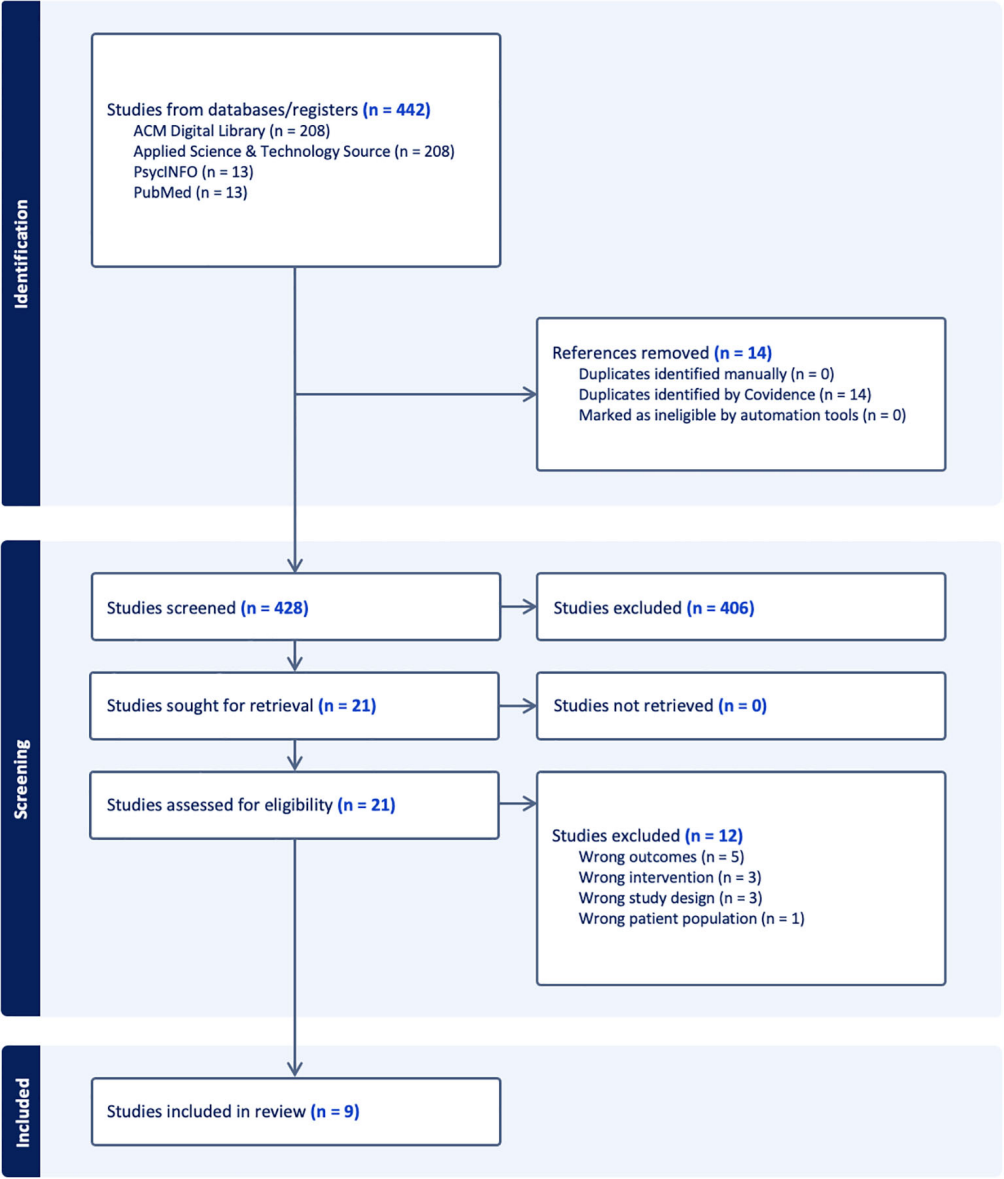
PRISMA flow diagram. PRISMA flow diagram (20).

The systematic search yielded 442 articles across four databases: ACM Digital Library (208), Applied Science & Technology Source (208), PsycINFO (13), and PubMed (13). After removing 14 duplicates, 428 records underwent title/abstract screening, excluding 406 irrelevant studies. Full-text review of 21 articles yielded 9 eligible studies for final analysis (**Figure 1**: PRISMA flowchart).

The nine included studies, encompassing 1,082 participants (sample sizes: 42–250) and eight distinct chatbots. Interventions ranged from single-use sessions (12 minutes) to 6-week programs, with most focusing on anxiety, depression, or well-being.

Eight studies (89%) reported statistically significant improvements in at least one mental health outcome (**Table 2**): (2, 6, 8, 9, 11, 22–24).

- Anxiety/Depression Reduction: Five RCTs (Level I evidence) demonstrated symptom reductions using validated tools (PHQ-9, GAD-7). For example, Woebot (8) reduced depression scores by 22% (PHQ-9: Δ = −3.16, p < 0.05) in two weeks.
- Well-Being Improvements: Jibo (22) increased psychological well-being scores by 22% (RPWS: 21.28 → 25.96, p < 0.01).
- Academic Stress: ARU (24) reduced academic stress metrics (Working Alliance Inventory: Δ = −1.71, p = 0.03).

**Table 2:** Overall effectiveness of chatbot.

Article	Level of evidence	PEDro score	Effectiveness	Effect sizes
(21)	I (RCT)	3 (Poor)	↔ (Neutral)	**Well-being Trial 1:** *d* = -0.05 (very small) **Well-being Trial 2:** *d* = -0.15 (small)
(8)	I (RCT)	6 (Good)	+	**Anxiety**: *d* = 0.37 (small) **Depression**: *d* = 0.44 (medium)
(2)	I (RCT)	7 (Good)	+	^c^ **Anxiety:** N/A **Depression**: *d* = 0.68 (medium)
(6)	II	1 (Poor)	+	^a^ **Anxiety**: *d* = -0.36 (small) **Stress**: *d* = -0.36 (small)
(22)	II	1 (Poor)	+	^a^ **Group 1** **Well-being**: *d* = 2.79 (very large) **Mood:** *d* = 0.29 (small) **Readiness:** *d* = 0.48 (medium) **Group 2** **Well-being**: *d* = 1.80 (large) **Mood:** *d* = 1.05 (large) **Readiness:** *d* = 1.03 (large)
(9)	II	2 (Poor)	+	^b^N/A
(23)	I (RCT)	3 (Poor)	+	**Anxiety**: *d* = 0.50 (medium) **Depression**: *d* = 0.09 (very small)
(11)	I (RCT)	5 (Fair)	+	**Anxiety**: *d* = 0.30 (small) **Depression**: *d* = 0.83 (large)
(24)	II	5 (Fair)	+	^a^ **Stress** **Group 1:** *d* = -0.33 (small) **Group 2:** *d* = -0.38 (small) **Group 3:** *d* = -0.72 (large)

a within group effect size only due to absence of control group.

b none due to missing mean and SD values.

c only depression effect size included in study. Effect sizes for shorter intervention and anxiety could not be calculated due to the absence of mean and SD values.

Group differences were interpreted per Cohen's guidelines (e.g., d < 0.2: very weak; 0.2–0.5: weak; 0.5–0.8: medium; > 0.8: large) for clinical relevance (25).

One study (Mind Tutor; 21) showed no significant changes in well-being (SWEMWBS: Δ = +0.04, p = 0.62), potentially due to brief intervention duration (6 weeks) or lack of personalized feedback.

As for the Level of Evidence, there were five Level I RCTs and four Level II quasi-experimental studies were included. There were two studies with good quality (PEDro scores 6–8): 2, 8. Two studies were rated fair (PEDro scores 4-5): 11, 24. The remaining studies were rated poor (PEDro scores <5), limited by small samples or lack of control groups. Higher-quality studies (PEDro ≥6) consistently supported chatbot efficacy, whereas lower-scoring studies (e.g., 6; PEDro = 1) showed smaller effect sizes.

For the Chatbot design and intervention delivery (**Table 3**), we noticed that effective interventions shared those key features: 1) CBT integration: Woebot and XiaoNan used structured CBT modules (e.g., mood tracking, cognitive restructuring). 2) Personalization: ARU incorporated cultural adaptation for Indian students, improving adherence. 3) Optimal delivery frequency: Daily interactions (e.g., Tess; 2) correlated with greater engagement (usage rate: 78% vs. 52% in biweekly groups). In contrast, passive apps (Mind Tutor) with static content showed minimal impact, and brief interventions (<2 weeks; e.g., Gloomy; 9) had transient effects.

**Table 3:** Chatbot interventions.

Article	Chatbot	Duration	Key features	Intervention protocol
(21)	Mind Tutor	6 weeks	Academic + well-being integration; mindfulness, goal-setting	6-week app access: topic-specific modules (anxiety, mood, academics) + chatbot guidance
(8)	Woebot	2 weeks	CBT-focused; mood tracking, goal setting, emojis	Daily 5–10-minute CBT conversations via messenger app
(2)	Tess	2–4 weeks	Integrative mental health support (psychoeducation, reminders)	Group 1: Daily check-ins (2 weeks); Group 2: Biweekly check-ins (4 weeks)
(6)	Atena	4 weeks	AI-driven; CBT, mindfulness, psychoeducation	10-minute sessions, twice weekly (8 total); personalized schedule
(22)	Jibo	1 week	Social robot; expressive movements; positive psychology	5-minute daily sessions: goal-setting, mood reflection
(9)	Gloomy	3 weeks	Social media-integrated (Facebook); symptom self-reflection	Evening posts; user comments triggered gratitude/emotional support responses
(23)	Tess	8 weeks	AI-driven; empathetic text/emojis; mood tracking	Daily FB Messenger check-ins (weeks 1–4), then biweekly (weeks 5–8)
(11)	XiaoNan	16 weeks	CBT-based; depression assessment; empathetic responses	Daily emotion logging + random conversations; automated CBT templates
(24)	ARU	1 session	Culturally adapted (India); academic stress management (diet, exercise, social)	Single 12-minute session; tailored behavioral advice

**Figure 2:**
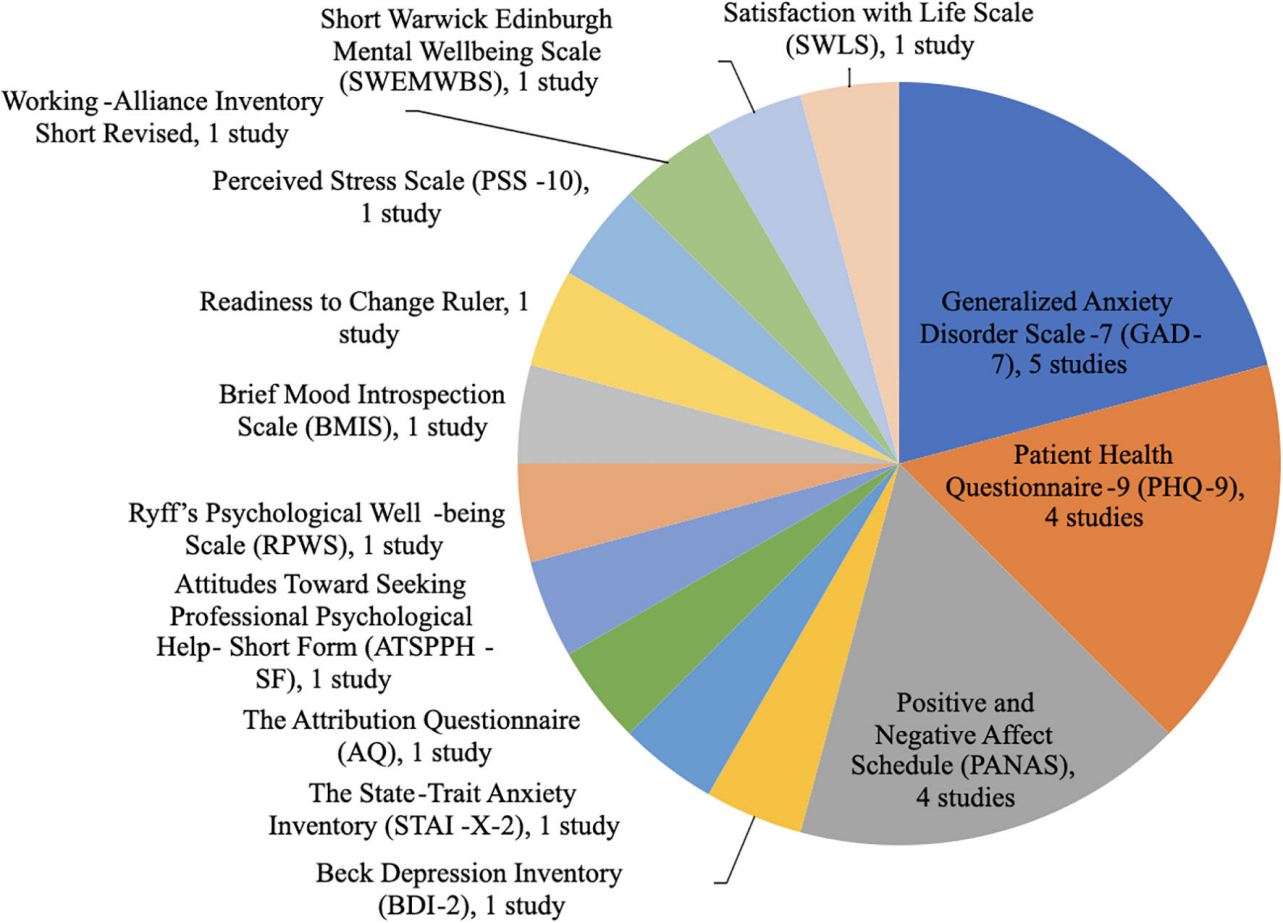
Utilization of assessment tools to measure chatbot effectiveness for mental health.

A total of 14 different outcome measures were used across the nine studies as shown in **Table 4**, with three predominating (**Figure 2**): GAD-7 (5 studies): Detected anxiety reductions (e.g., 6: Δ = −1.20, p = 0.04). PHQ-9 (4 studies): Tracked depression improvements (e.g., 11: chatbot Δ = −5.25 vs. control Δ = −2.98, p < 0.001). PANAS (4 studies): Captured mood shifts (e.g., 8: negative affect Δ = −1.26 vs. control Δ = +1.21).

**Table 4:** Outcome measures.

Article	Condition	Outcome measures	Sample size	Key results (pre → post)
(21)	Well-being	SWEMWBS, SWLS, PANAS-SF	177 (Trial 1), 250 (Trial 2)	**Trial 1**: Minimal change **Trial 2**: Well-being ↔ (3.12→3.16)
(8)	Anxiety & Depression	PHQ-9, GAD-7, PANAS	70 (34 EG, 36 CG)	**Woebot (EG)**: Depression ↓22% (14.30→11.14) **Control**: No significant change
(2)	Anxiety & Depression	PHQ-9, GAD-7, PANAS	74 (24 EG1, 26 EG2, 24 CG)	**Daily Check-ins (EG1)**: Greater symptom reduction vs. biweekly (EG2) and control (CG)
(6)	Stress & Anxiety	GAD-7, PSS-10	71	**Anxiety**: 10.49 → 9.29 (↓13%) **Stress**: 22.49 → 20.83 (↓7%)
(22)	Well-being	RPWS, BMIS	42	**Well-being**: ↑22% (21.28→25.96) **Mood**: ↑12% (6.80→7.63)
(9)	Anxiety, Depression, Stigma	BDI-2, STAI-X-2, AQ	55	**Anxiety**: Median ↓10% (42→38) **Depression**: Median ↓60% (10→4)
(23)	Anxiety & Depression	PHQ-9, GAD-7	73 (39 EG, 34 CG)	**Chatbot Group**: Anxiety ↓16% (15.59→13.04) **Control**: Anxiety ↑6% (15.35→16.26)
(11)	Depression & Anxiety	PHQ-9, GAD-7, PANAS	83 (41 EG, 42 CG)	**Chatbot Group**: Anxiety ↓9% (15.59→14.23) Depression ↓41% (13.17→7.92) **Control**: Minimal change
(24)	Academic Stress	Working Alliance Inventory	61	**Group 1:** 7.65 → 6.91 (↓10%) **Group 2**: 7.28 → 6.43 (↓12%) **Group 3**: 7.59 → 5.88 (↓23%)

Group differences were interpreted per Cohen's guidelines (e.g., d < 0.2: very weak; 0.2–0.5: weak; 0.5–0.8: medium; > 0.8: large) for clinical relevance (25).

The most used instruments were the Generalized Anxiety Disorder Scale-7 (GAD-7), Patient Health Questionnaire-9 (PHQ-9), and Positive and Negative Affect Schedule (PANAS) as shown in **Figure 2**.

Five studies used the GAD-7 (2, 6, 8, 11, 23). This is a seven-item self-report scale to assess anxiety symptoms over the past two weeks, it uses a four-point Likert scale, ranging from 0 (not at all) to 3 (nearly every day). Four studies used the PHQ-9 (2, 8, 11, 23). This is a nine-item self-report questionnaire that assesses the frequency and severity of depressive symptoms within the previous two weeks. Four studies used the PANAS (2, 8, 11, 21). This is a 20-item self-report measure of current positive and negative effects. All other instruments reported in **Figure 2** were only used by one study.

## 4 Discussion

### 4.1 Principal results

This systematic rapid review synthesizes evidence from nine studies evaluating chatbots for mental health and well-being in college students. Most interventions (8/9 studies) demonstrated efficacy in reducing anxiety, depression, or improving well-being, particularly when grounded in CBT and designed for sustained engagement (≥2 weeks). Notably, chatbots with daily interactions (e.g., Woebot, Tess) achieved greater symptom reduction than shorter or less frequent interventions. However, variability in study quality (only two “good” PEDro scores) and heterogeneity in outcome measures limits definitive conclusions.

This systematic review provides potential guidelines for future chatbots for mental health and well-being interventions. For instance, chatbots employing structured CBT techniques (e.g., mood tracking, cognitive restructuring) showed consistent efficacy, aligning with evidence that skill-based interventions outperform passive psychoeducation (8, 11). Interventions with daily check-ins (e.g., 2) outperformed those with biweekly interactions, suggesting that consistency reinforces habit formation and therapeutic alliance. In addition, tailored designs, such as ARU’s culturally resonant interface for Indian students (24), improved engagement and adherence. The predominance of PHQ-9 and GAD-7 across studies supports their utility as gold-standard measures. However, well-being metrics (e.g., SWEMWBS) were underutilized, and mixed results in this domain (e.g., Mind Tutor vs. Jibo) highlight the need for validated, context-specific tools.

Our findings align with broader reviews (26, 27) affirming chatbots’ potential as scalable mental health tools. However, this review uniquely identifies college students as a population benefiting from chatbots’ 24/7 availability and stigma- reducing anonymity, critical factors in high-stress academic environments. Notably, unlike prior reviews focused on general populations, we identified academic stress as a distinct target for chatbot interventions, with ARU demonstrating feasibility in this domain (24).

Key limitations regarding chatbots for the target population identified in the included studies are that chatbots lack the capacity to escalate emergencies, risking under-treatment of severe cases., and over-reliance on chatbots may delay help-seeking from human providers, necessitating hybrid models (27).

This rapid review is the first to synthesize evidence on AI-driven chatbot interventions specifically for college students, who are facing unique mental health challenges due to academic stress, transitional life stages, and limited access to traditional care. By identifying effective design features, such as CBT integration, daily interactions, and cultural personalization, this review offers practical guidelines for developing scalable, stigma-free interventions tailored to university settings. For instance, chatbots like Woebot and Tess demonstrate significant reductions in anxiety (GAD-7, p=0.04) and depression (PHQ-9, p<0.001), suggesting their potential as adjuncts to overburdened counseling services. Additionally, our findings highlight critical gaps, such as the need for emergency response protocols and standardized outcome measures, providing a roadmap for future research and development. These insights are particularly timely given the post-COVID-19 mental health crisis, offering universities actionable strategies to integrate chatbots into hybrid care models, especially during high-stress periods like exams.

### 4.2 Limitations

Limitations of the review include the number of studies found and reviewed. We acknowledge that a full systematic review would provide a more exhaustive synthesis. However, our intent with this rapid review was not to replace a systematic review but to serve as an initial, timely appraisal of the literature that could guide future research, including a full systematic review where appropriate. After the initial full-text screening, several more studies were excluded due to the lack of post measures. Small sample sizes (e.g., Jibo: n=42) and high dropout rates (e.g., 23: 61%) reduce generalizability. Additionally, inconsistent intervention durations (1 week–6 months) and engagement protocols complicate cross-study comparisons. Lastly, positive results may be overrepresented, as null findings (e.g., Mind Tutor) are less likely to be published.

### 4.3 Comparison with prior work

Similar reviews have validated the findings in this review. In a systematic review performed by Abd-Alrazaq et al. (26), the authors agreed that chatbots do have the potential to improve mental health. However, the review could not definitively conclude this due to similar limitations such as studies lacking certain measures and certain studies showing no statistically significant difference between chatbots and other interventions. In an exploratory observation conducted by Haque and Rubya (27), the authors also found chatbots to have the potential to improve mental health. Positive components found that users enjoyed having a virtual companion that is available 24/7 and provides a judgment-free space. The study, however, noted that all these beneficial factors can make it easy for an individual to become too attached to the chatbot. Another finding identified in the study was that the chatbots were not able to identify a crisis. This has implications for future research to assess the safety of chatbots. It is important to note that both studies explored chatbot use to improve mental health in a general population, and not specifically college students.

## 5 Conclusions

Chatbots represent a promising, scalable solution to address the mental health crisis among college students, particularly when integrating evidence-based therapies like CBT and prioritizing frequent, personalized engagement. While this review underscores their potential to reduce anxiety, depression, and academic stress, critical gaps remain: 1) Consensus on core outcome measures (e.g., PHQ-9, GAD-7) and intervention duration (≥2 weeks) is needed. 2) Future chatbots must incorporate emergency responses to students with severe symptoms and referrals to human providers. Our findings support piloting chatbots as adjuncts, not replacements to traditional counseling, particularly during peak stress periods (e.g.,exams).
